# Supplementary material for: Abiotic formation of condensed carbonaceous matter in the hydrating oceanic crust
Source: Nat Commun. 2018 Nov 28;9:5049. doi: 10.1038/s41467-018-07385-6 (PMC6261978; doi:10.1038/s41467-018-07385-6)
Supplement: Supplementary file 1 — Supplementary Information [file 41467_2018_7385_MOESM1_ESM.pdf]

Supplementary Information

Abiotic formation of condensed carbonaceous matter in the hydrating oceanic crust

Marie Catherine Sforna[1]*, Daniele Brunelli[1,2], Céline Pisapia[3], Valerio Pasini[1,3], Daniele Malferrari[1], and Bénédicte Ménez[3]

[1] Dipartimento di Scienze Chimiche e Geologiche, Università di Modena e Reggio Emilia. Via Campi, 103 – 41125 Modena, Italy. [2] Istituto di Scienze del Mare – ISMAR-CNR. Via Gobetti, 101 – 49100 Bologna, Italy. [3] Institut de Physique du Globe de Paris, Sorbonne Paris Cité, Université Paris Diderot, CNRS UMR 7154. 1 rue Jussieu – 75238 Paris cedex 5, France. [4]Synchrotron SOLEIL. L'orme des merisiers, Saint-Aubin BP48 – 91192 Gif-sur-Yvette cedex, France.

*e-mail: marie.sforna@unimore.it

This Supplementary Information contains 16 Supplementary Tables, 2 Supplementary Figures and 11 References.

Supplementary Tables:

Mesh Serpentine

	SiO_2	TiO_2	Al_2O_3	Cr_2O_3	$(FeO)_T$	MnO	MgO	NiO	CaO	Na_2O	TOTAL	Mg#
Mesh core Zone 1	41.59	0.02	1.22	0.00	3.77	0.00	37.95	0.56	0.10	0.04	85.26	88.57
	41.39	0.00	1.23	0.00	4.47	0.00	37.29	0.55	0.14	0.04	85.12	86.52
	41.07	0.02	1.46	0.01	3.72	0.01	37.30	0.47	0.15	0.04	84.25	88.51
	41.30	0.00	1.24	0.00	3.74	0.00	37.47	0.53	0.12	0.04	84.44	88.52
	41.23	0.01	1.27	0.00	3.76	0.00	37.83	0.55	0.12	0.03	84.79	88.57
Mean	*41.32*	*0.01*	*1.28*	*0.00*	*3.89*	*0.00*	*37.57*	*0.53*	*0.13*	*0.04*	*84.77*	*88.14*
Standard deviation (1 σ)	*0.19*	*0.01*	*0.10*	*0.00*	*0.32*	*0.00*	*0.31*	*0.03*	*0.02*	*0.00*	*1.00*	*0.91*
Mesh core Zone 2	41.27	0.00	1.37	0.00	4.71	0.00	37.01	0.46	0.14	0.04	85.01	85.80
	41.47	0.01	1.30	0.00	4.38	0.00	37.55	0.48	0.14	0.03	85.36	86.84
	41.52	0.02	1.41	0.01	4.41	0.00	37.07	0.41	0.12	0.03	85.00	86.62
Mean	*41.42*	*0.01*	*1.36*	*0.00*	*4.50*	*0.00*	*37.21*	*0.45*	*0.13*	*0.03*	*85.12*	86.42
Standard deviation (1 σ)	*0.13*	*0.01*	*0.06*	*0.00*	*0.18*	*0.00*	*0.29*	*0.03*	*0.01*	*0.00*	*0.73*	*0.16*
Mesh rim Zone 1	41.86	0.01	0.42	0.01	2.91	0.02	39.25	0.29	0.12	0.01	84.90	91.20
	42.55	0.01	0.41	0.00	2.82	0.00	39.47	0.25	0.06	0.03	85.60	91.51
	42.47	0.00	0.31	0.01	2.53	0.00	39.68	0.41	0.05	0.03	85.49	92.36
	42.88	0.00	0.35	0.01	2.55	0.00	39.57	0.28	0.06	0.03	85.74	92.26
Mean	*42.44*	*0.00*	*0.37*	*0.01*	*2.70*	*0.00*	*39.49*	*0.31*	*0.07*	*0.03*	*85.43*	*91.83*
Standard deviation (1 σ)	*0.42*	*0.01*	*0.05*	*0.00*	*0.19*	*0.01*	*0.18*	*0.07*	*0.03*	*0.01*	*0.37*	*0.57*

MESH	SiO_2	TiO_2	Al_2O_3	Cr_2O_3	$(FeO)_T$	MnO	MgO	NiO	CaO	Na_2O	TOTAL	Mg#
Mean cores	*41.36*	*0.01*	*1.31*	*0.00*	*4.12*	*0.00*	*37.43*	*0.50*	*0.13*	*0.04*	*84.90*	*91.83*
Standard deviation (1 σ)	*0.17*	*0.01*	*0.09*	*0.00*	*0.41*	*0.00*	*0.34*	*0.05*	*0.02*	*0.01*	*0.39*	*1.16*
Mean rims	*42.44*	*0.00*	*0.37*	*0.01*	*2.70*	*0.00*	*39.49*	*0.31*	*0.07*	*0.03*	*85.43*	*87.49*
Standard deviation (1 σ)	*0.42*	*0.01*	*0.05*	*0.00*	*0.19*	*0.01*	*0.18*	*0.07*	*0.03*	*0.01*	*0.37*	*0.57*

Supplementary Table 1. Microprobe analyses (%Wt) and average compositions measured on the serpentine constituting the cores and rims of the mesh structure of the Casale serpentinites. Analyses were performed in punctual mode. $(FeO)_T$ corresponds to total ferric and ferrous iron. The corresponding Mg# values (Mg/(Mg+Fe)) are also reported.

Bastite

	SiO_2	TiO_2	Al_2O_3	Cr_2O_3	$(FeO)_T$	MnO	MgO	NiO	CaO	Na_2O	TOTAL	Fe#
Bastite - Point 1	39.42	0.14	3.09	0.81	5.58	0.05	34.78	0.16	0.17	0.04	84.23	86.17
Bastite - Point 2	39.38	0.17	3.05	0.81	5.69	0.05	34.55	0.17	0.17	0.05	84.08	85.85
Bastite - Point 3	39.56	0.18	2.96	0.81	5.45	0.05	34.76	0.22	0.15	0.05	84.20	86.44
Bastite - Point 4	39.74	0.17	2.81	0.74	5.75	0.04	35.04	0.20	0.16	0.05	84.71	85.90

BASTITE	SiO_2	TiO_2	Al_2O_3	Cr_2O_3	$(FeO)_T$	MnO	MgO	NiO	CaO	Na_2O	TOTAL	Fe#
Mean	39.53	0.17	2.98	0.79	5.62	0.05	34.78	0.19	0.16	0.04	84.31	86.09
Standard deviation (1 σ)	0.16	0.02	0.12	0.03	0.13	0.00	0.20	0.03	0.01	0.01	0.28	0.27

Supplementary Table 2. Microprobe analyses (%Wt) and average compositions measured on the bastite of the Casale serpentinites. Analyses were performed in punctual mode. $(FeO)_T$ corresponds to total ferric and ferrous iron. The calculated Fe# values ($(FeO)_T/(Mg+(FeO)_T)$) are also reported.

Spinel cores

Raw data

	SiO_2	TiO_2	Al_2O_3	Cr_2O_3	$(FeO)_T$	MnO	MgO	NiO	CaO	Na_2O	TOTAL
Spl core - Point 1	0.08	0.44	41.62	24.62	16.31	1.34	16.98	0.36	0.03	0.02	101.74
Spl core - Point 2	0.05	0.45	41.81	24.84	15.93	1.37	17.09	0.37	0.00	0.01	101.91
Spl core - Point 3	0.13	0.48	41.83	25.12	15.29	1.42	16.57	0.30	0.07	0.09	101.14
Spl core - Point 4	0.08	0.41	41.36	25.06	15.58	1.43	16.80	0.47	0.05	0.00	101.19
Spl core - Map1	0.67	0.75	37.75	26.40	13.73	2.02	17.20	3.09	0.00	0.13	101.74
	0.00	0.50	38.47	25.23	18.64	1.62	17.71	3.08	0.12	0.26	105.62
	0.51	0.75	40.15	24.36	18.75	1.52	17.25	1.55	0.00	0.25	105.09

Normalization after recalculation Fe^{3+}/Fe^{2+}

Spl core - Point 1		SiO_2	TiO_2	Al_2O_3	Cr_2O_3	Fe_2O_3	FeO	MnO	MgO	TOTAL	Cr #	Mg #
	%Wt	0.08	0.44	41.62	24.62	4.44	12.32	1.34	16.98	101.83	28.41	71.07
		Si	Ti	Al	Cr	Fe^{3+}	Fe^{2+}	Mn	Mg	Fe^{3+}/Fe^{2+}	Fe #	Fe^{3+} #
	Atoms	0.00	0.01	1.35	0.54	0.09	0.28	0.03	0.70	0.32	28.93	4.65
Spl core - Point 2		SiO_2	TiO_2	Al_2O_3	Cr_2O_3	Fe_2O_3	FeO	MnO	MgO	TOTAL	Cr #	Mg #
	%Wt	0.05	0.45	41.81	24.84	4.19	12.16	1.37	17.09	101.95	28.50	71.47
		Si	Ti	Al	Cr	Fe^{3+}	Fe^{2+}	Mn	Mg	Fe^{3+}/Fe^{2+}	Fe #	Fe^{3+} #
	Atoms	0.00	0.01	1.35	0.54	0.09	0.28	0.03	0.70	0.31	28.53	4.38
Spl core - Point 3		SiO_2	TiO_2	Al_2O_3	Cr_2O_3	Fe_2O_3	FeO	MnO	MgO	TOTAL	Cr #	Mg #
	%Wt	0.13	0.48	41.83	25.12	2.77	12.80	1.42	16.57	101.12	28.72	69.78
		Si	Ti	Al	Cr	Fe^{3+}	Fe^{2+}	Mn	Mg	Fe^{3+}/Fe^{2+}	Fe #	Fe^{3+} #
	Atoms	0.00	0.01	1.36	0.55	0.06	0.30	0.03	0.68	0.19	30.22	2.93
Spl core - Point 4		SiO_2	TiO_2	Al_2O_3	Cr_2O_3	Fe_2O_3	FeO	MnO	MgO	TOTAL	Cr #	Mg #
	%Wt	0.08	0.41	41.36	25.06	3.76	12.20	1.43	16.80	101.10	28.89	71.03
		Si	Ti	Al	Cr	Fe^{3+}	Fe^{2+}	Mn	Mg	Fe^{3+}/Fe^{2+}	Fe #	Fe^{3+} #
	Atoms	0.00	0.01	1.35	0.55	0.08	0.28	0.03	0.69	0.28	28.97	3.94

Normalization after recalculation Fe^{3+}/Fe^{2+}

Spl core - Map 1 - Point 1		SiO_2	TiO_2	Al_2O_3	Cr_2O_3	Fe_2O_3	FeO	MnO	MgO	TOTAL	Cr #	Mg #
	%Wt	0.67	0.75	37.75	26.40	3.25	10.81	2.02	17.20	98.85	31.94	73.94
		Si	Ti	Al	Cr	Fe^{3+}	Fe^{2+}	Mn	Mg	Fe^{3+}/Fe^{2+}	Fe #	Fe^{3+} #
	Atoms	0.02	0.02	1.27	0.59	0.07	0.26	0.05	0.73	0.27	26.06	3.61
Spl core - Map 1 - Point 2		SiO_2	TiO_2	Al_2O_3	Cr_2O_3	Fe_2O_3	FeO	MnO	MgO	TOTAL	Cr #	Mg #
	%Wt	0.00	0.50	38.47	25.23	8.83	10.69	1.62	17.71	103.05	30.55	74.71
		Si	Ti	Al	Cr	Fe^{3+}	Fe^{2+}	Mn	Mg	Fe^{3+}/Fe^{2+}	Fe #	Fe^{3+} #
	Atoms	0.00	0.01	1.25	0.55	0.18	0.25	0.04	0.73	0.74	25.29	9.24
Spl core - Map 1 - Point 3		SiO_2	TiO_2	Al_2O_3	Cr_2O_3	Fe_2O_3	FeO	MnO	MgO	TOTAL	Cr #	Mg #
	%Wt	0.51	0.75	40.15	24.36	6.42	12.97	1.52	17.25	103.93	28.92	70.33
		Si	Ti	Al	Cr	Fe^{3+}	Fe^{2+}	Mn	Mg	Fe^{3+}/Fe^{2+}	Fe #	Fe^{3+} #
	Atoms	0.01	0.02	1.29	0.52	0.13	0.30	0.03	0.70	0.45	29.67	6.76

Spinel Cores	SiO_2	TiO_2	Al_2O_3	Cr_2O_3	Fe_2O_3	FeO	MnO	MgO	TOTAL	Fe^{3+}/Fe^{2+}	Cr#	Mg#	Fe#	Fe^{3+} #
Mean	0.22	0.54	40.43	25.09	4.81	11.99	1.53	17.09	101.69	0.37	29.42	71.76	28.24	5.07
Standard deviation (1 σ)	0.26	0.15	1.69	0.65	2.12	0.90	0.23	0.36	1.62	0.18	1.32	1.85	1.85	2.20

Supplementary Table 3. Microprobe analyses (%Wt) measured on spinel (spl) cores of the Casale serpentinites and number of atoms normalized for 4 oxygens. Analyses were performed in punctual mode and in mapping mode. $(FeO)_T$ corresponds to total ferric and ferrous iron. Normalized analyses after Fe^{3+}/Fe^{2+} recalculation are also reported along with Fe^{3+}/Fe^{2+}, Cr# (Cr/(Cr+Al)), Mg#, Fe# and Fe^{3+}# ($100 \times Fe^{3+}/(Fe^{3+}+Cr+Al)$) values.

Ferritchromite rims

Raw data

	SiO_2	TiO_2	Al_2O_3	Cr_2O_3	$(FeO)_T$	MnO	MgO	NiO	CaO	Na_2O	TOTAL
Ftc - Point 1	3.90	0.35	34.39	22.82	18.81	1.23	14.28	0.34	2.54	0.00	98.67
Ftc - point 2	6.41	0.49	34.91	23.99	15.29	1.45	14.38	0.11	5.18	0.01	102.22
Ftc - point 3	5.71	0.38	35.09	24.40	16.74	1.25	15.93	0.09	2.01	0.02	101.63
Ftc - Map1	7.55	0.48	6.90	24.82	33.90	2.42	12.49	3.02	0.45	0.15	92.17
	12.35	0.78	5.77	26.27	37.72	1.52	10.05	1.51	0.45	0.30	96.71
	11.05	0.59	5.70	23.71	47.94	1.76	11.09	1.51	0.44	0.00	103.79
	7.33	0.41	6.81	26.55	34.20	1.73	9.94	0.00	0.55	0.46	87.98
	10.74	0.95	8.73	25.83	42.41	2.00	10.52	0.00	0.67	0.76	102.62
	8.38	0.83	5.52	24.87	44.55	2.20	10.03	0.00	0.55	0.31	97.24
	8.58	0.71	6.70	26.56	44.45	1.44	10.13	1.50	0.33	0.00	100.40
	10.11	0.54	9.44	26.77	31.93	2.12	9.61	0.00	0.45	0.30	91.28
	7.97	0.65	6.85	25.05	36.33	2.26	9.34	1.51	0.44	0.16	90.55
Ftc - Map 2	8.83	0.83	6.10	26.38	31.83	2.00	8.71	0.00	0.44	0.00	85.12
	7.35	0.59	7.02	26.46	39.86	2.30	10.24	1.51	0.00	0.31	95.64
	10.37	0.83	4.00	23.95	40.09	1.89	10.66	0.00	0.33	0.15	92.27
	11.16	0.66	4.99	24.76	36.64	1.46	11.85	0.00	0.33	0.30	92.15
	10.25	0.71	6.02	25.89	50.35	2.36	11.07	0.00	0.66	0.93	108.25
	10.42	0.95	5.03	23.51	40.88	2.20	10.13	3.01	0.67	0.00	96.80
	6.23	0.71	7.95	26.77	36.33	1.87	7.08	0.00	0.44	0.62	88.01
	7.00	1.05	3.40	24.97	52.40	1.54	10.45	0.00	0.33	0.32	101.45
	9.76	0.83	5.64	25.46	37.83	1.79	10.70	0.00	0.78	0.15	92.93
	9.62	0.71	5.94	26.22	28.32	1.41	10.33	0.00	0.00	0.15	82.70
	10.11	0.48	6.65	25.62	42.41	1.94	12.30	0.00	0.78	0.46	100.75
	9.48	0.66	6.11	24.58	28.18	2.17	9.23	1.51	0.45	0.15	82.51
	13.86	0.48	4.14	25.94	40.08	1.46	10.96	1.51	0.78	0.15	99.37

Supplementary Table 4. Microprobe analyses (%Wt) measured on ferritchromite (Ftc) rims of the Casale serpentinites and the number of atoms normalized for 4 oxygens. Analyses were performed in punctual mode and in mapping mode. $(FeO)_T$ corresponds to total ferric and ferrous iron. Normalized analyses after Fe^{3+}/Fe^{2+} recalculation (based on spinel structure[51]) are also reported along with Fe^{3+}/Fe^{2+} and Fe^{3+}# values. Table continues on the following pages.

Supplementary Table 4 (continued)

Normalization after recalculation Fe^{3+}/Fe^{2+}

Ftc - Point 1		SiO_2	TiO_2	Al_2O_3	Cr_2O_3	Fe_2O_3	FeO	MnO	MgO	TOTAL	
	%Wt	3.90	0.35	34.39	22.82	0.40	18.44	1.23	14.28	95.82	
		Si	Ti	Al	Cr	Fe^{3+}	Fe^{2+}	Mn	Mg	Fe^{3+}/Fe^{2+}	Fe^{3+}#
	Atoms	0.12	0.01	1.21	0.54	0.01	0.46	0.03	0.63	0.02	51.40
Ftc - Point 2		SiO_2	TiO_2	Al_2O_3	Cr_2O_3	Fe_2O_3	FeO	MnO	MgO	TOTAL	
	%Wt	6.41	0.49	34.91	23.99	0.00	15.29	1.45	14.38	96.92	
		Si	Ti	Al	Cr	Fe^{3+}	Fe^{2+}	Mn	Mg	Fe^{3+}/Fe^{2+}	Fe^{3+}#
	Atoms	0.19	0.01	1.21	0.56	0.00	0.37	0.04	0.63	0.00	0.00
Ftc - Point 3		SiO_2	TiO_2	Al_2O_3	Cr_2O_3	Fe_2O_3	FeO	MnO	MgO	TOTAL	
	%Wt	5.71	0.38	35.09	24.40	0.00	16.74	1.25	15.93	99.50	
		Si	Ti	Al	Cr	Fe^{3+}	Fe^{2+}	Mn	Mg	Fe^{3+}/Fe^{2+}	Fe^{3+}#
	Atoms	0.16	0.01	1.18	0.55	0.00	0.40	0.03	0.68	0.00	0.00

Ftc - Map 1 - Point 1		SiO_2	TiO_2	Al_2O_3	Cr_2O_3	Fe_2O_3	FeO	MnO	MgO	TOTAL	
	%Wt	7.55	0.48	6.90	24.82	17.12	18.50	2.42	12.49	89.69	
		Si	Ti	Al	Cr	Fe^{3+}	Fe^{2+}	Mn	Mg	Fe^{3+}/Fe^{2+}	Fe^{3+}#
	Atoms	0.27	0.01	0.29	0.70	0.46	0.55	0.07	0.66	0.83	31.70
Ftc - Map 1 - Point 2		SiO_2	TiO_2	Al_2O_3	Cr_2O_3	Fe_2O_3	FeO	MnO	MgO	TOTAL	
	%Wt	12.35	0.78	5.77	26.27	7.22	31.22	1.52	10.05	95.17	
		Si	Ti	Al	Cr	Fe^{3+}	Fe^{2+}	Mn	Mg	Fe^{3+}/Fe^{2+}	Fe^{3+}#
	Atoms	0.42	0.02	0.23	0.71	0.18	0.89	0.04	0.51	0.21	16.46
Ftc - Map 1 - Point 3		SiO_2	TiO_2	Al_2O_3	Cr_2O_3	Fe_2O_3	FeO	MnO	MgO	TOTAL	
	%Wt	11.05	0.59	5.70	23.71	19.84	30.09	1.76	11.09	103.82	
		Si	Ti	Al	Cr	Fe^{3+}	Fe^{2+}	Mn	Mg	Fe^{3+}/Fe^{2+}	Fe^{3+}#
	Atoms	0.35	0.01	0.21	0.59	0.47	0.79	0.05	0.52	0.59	36.96
Ftc - Map 1 - Point 4		SiO_2	TiO_2	Al_2O_3	Cr_2O_3	Fe_2O_3	FeO	MnO	MgO	TOTAL	
	%Wt	7.33	0.41	6.81	26.55	13.38	22.16	1.73	9.94	88.31	
		Si	Ti	Al	Cr	Fe^{3+}	Fe^{2+}	Mn	Mg	Fe^{3+}/Fe^{2+}	Fe^{3+}#
	Atoms	0.27	0.01	0.30	0.77	0.37	0.68	0.05	0.55	0.54	25.75
Ftc - Map 1 - Point 5		SiO_2	TiO_2	Al_2O_3	Cr_2O_3	Fe_2O_3	FeO	MnO	MgO	TOTAL	
	%Wt	10.74	0.95	8.73	25.83	12.91	30.79	2.00	10.52	102.48	
		Si	Ti	Al	Cr	Fe^{3+}	Fe^{2+}	Mn	Mg	Fe^{3+}/Fe^{2+}	Fe^{3+}#
	Atoms	0.34	0.02	0.32	0.64	0.31	0.81	0.05	0.50	0.38	24.03
Ftc - Map 1 - Point 6		SiO_2	TiO_2	Al_2O_3	Cr_2O_3	Fe_2O_3	FeO	MnO	MgO	TOTAL	
	%Wt	8.38	0.83	5.52	24.87	20.36	26.23	2.20	10.03	98.42	
		Si	Ti	Al	Cr	Fe^{3+}	Fe^{2+}	Mn	Mg	Fe^{3+}/Fe^{2+}	Fe^{3+}#
	Atoms	0.28	0.02	0.22	0.66	0.51	0.74	0.06	0.50	0.70	36.93
Ftc - Map 1 - Point 7		SiO_2	TiO_2	Al_2O_3	Cr_2O_3	Fe_2O_3	FeO	MnO	MgO	TOTAL	
	%Wt	8.58	0.71	6.70	26.56	18.45	27.85	1.44	10.13	100.42	
		Si	Ti	Al	Cr	Fe^{3+}	Fe^{2+}	Mn	Mg	Fe^{3+}/Fe^{2+}	Fe^{3+}#
	Atoms	0.28	0.02	0.26	0.69	0.46	0.76	0.04	0.50	0.60	32.46
Ftc - Map 1 - Point 8		SiO_2	TiO_2	Al_2O_3	Cr_2O_3	Fe_2O_3	FeO	MnO	MgO	TOTAL	
	%Wt	10.11	0.54	9.44	26.77	5.01	27.43	2.12	9.61	91.03	
		Si	Ti	Al	Cr	Fe^{3+}	Fe^{2+}	Mn	Mg	Fe^{3+}/Fe^{2+}	Fe^{3+}#
	Atoms	0.35	0.01	0.39	0.74	0.13	0.80	0.06	0.50	0.16	10.45
Ftc - Map 1 - Point 9		SiO_2	TiO_2	Al_2O_3	Cr_2O_3	Fe_2O_3	FeO	MnO	MgO	TOTAL	
	%Wt	7.97	0.65	6.85	25.05	13.61	24.08	2.26	9.34	89.80	
		Si	Ti	Al	Cr	Fe^{3+}	Fe^{2+}	Mn	Mg	Fe^{3+}/Fe^{2+}	Fe^{3+}#
	Atoms	0.29	0.02	0.29	0.72	0.37	0.73	0.07	0.51	0.51	26.87

Supplementary Table 4 (continued)

Normalization after recalculation Fe^{3+}/Fe^{2+}

Ftc - Map 2 - Point 1		SiO_2	TiO_2	Al_2O_3	Cr_2O_3	Fe_2O_3	FeO	MnO	MgO	TOTAL	
	%Wt	8.83	0.83	6.10	26.38	7.42	25.16	2.00	8.71	85.42	
		Si	Ti	Al	Cr	Fe^{3+}	Fe^{2+}	Mn	Mg	Fe^{3+}/Fe^{2+}	Fe^{3+}#
	Atoms	0.34	0.02	0.27	0.79	0.21	0.80	0.06	0.49	0.27	16.60
Ftc - Map 2 - Point 2		SiO_2	TiO_2	Al_2O_3	Cr_2O_3	Fe_2O_3	FeO	MnO	MgO	TOTAL	
	%Wt	7.35	0.59	7.02	26.46	18.05	23.62	2.30	10.24	95.63	
		Si	Ti	Al	Cr	Fe^{3+}	Fe^{2+}	Mn	Mg	Fe^{3+}/Fe^{2+}	Fe^{3+}#
	Atoms	0.25	0.02	0.28	0.72	0.47	0.68	0.07	0.52	0.69	31.76
Ftc - Map 2 - Point 3		SiO_2	TiO_2	Al_2O_3	Cr_2O_3	Fe_2O_3	FeO	MnO	MgO	TOTAL	
	%Wt	10.37	0.83	4.00	23.95	15.25	26.37	1.89	10.66	93.31	
		Si	Ti	Al	Cr	Fe^{3+}	Fe^{2+}	Mn	Mg	Fe^{3+}/Fe^{2+}	Fe^{3+}#
	Atoms	0.36	0.02	0.17	0.66	0.40	0.77	0.06	0.56	0.52	32.66
Ftc - Map 2 - Point 4		SiO_2	TiO_2	Al_2O_3	Cr_2O_3	Fe_2O_3	FeO	MnO	MgO	TOTAL	
	%Wt	11.16	0.66	4.99	24.76	11.97	25.87	1.46	11.85	92.72	
		Si	Ti	Al	Cr	Fe^{3+}	Fe^{2+}	Mn	Mg	Fe^{3+}/Fe^{2+}	Fe^{3+}#
	Atoms	0.39	0.02	0.20	0.68	0.31	0.75	0.04	0.61	0.42	26.14
Ftc - Map 2 - Point 5		SiO_2	TiO_2	Al_2O_3	Cr_2O_3	Fe_2O_3	FeO	MnO	MgO	TOTAL	
	%Wt	10.25	0.71	6.02	25.89	22.38	30.21	2.36	11.07	108.89	
		Si	Ti	Al	Cr	Fe^{3+}	Fe^{2+}	Mn	Mg	Fe^{3+}/Fe^{2+}	Fe^{3+}#
	Atoms	0.31	0.02	0.21	0.62	0.51	0.77	0.06	0.50	0.67	37.92
Ftc - Map 2 - Point 6		SiO_2	TiO_2	Al_2O_3	Cr_2O_3	Fe_2O_3	FeO	MnO	MgO	TOTAL	
	%Wt	10.42	0.95	5.03	23.51	14.73	27.63	2.20	10.13	94.60	
		Si	Ti	Al	Cr	Fe^{3+}	Fe^{2+}	Mn	Mg	Fe^{3+}/Fe^{2+}	Fe^{3+}#
	Atoms	0.36	0.02	0.20	0.64	0.38	0.80	0.06	0.52	0.48	31.13
Ftc - Map 2 - Point 7		SiO_2	TiO_2	Al_2O_3	Cr_2O_3	Fe_2O_3	FeO	MnO	MgO	TOTAL	
	%Wt	6.23	0.71	7.95	26.77	12.17	25.38	1.87	7.08	88.17	
		Si	Ti	Al	Cr	Fe^{3+}	Fe^{2+}	Mn	Mg	Fe^{3+}/Fe^{2+}	Fe^{3+}#
	Atoms	0.23	0.02	0.35	0.80	0.34	0.80	0.06	0.40	0.43	23.07
Ftc - Map 2 - Point 8		SiO_2	TiO_2	Al_2O_3	Cr_2O_3	Fe_2O_3	FeO	MnO	MgO	TOTAL	
	%Wt	7.00	1.05	3.40	24.97	29.44	25.91	1.54	10.45	103.75	
		Si	Ti	Al	Cr	Fe^{3+}	Fe^{2+}	Mn	Mg	Fe^{3+}/Fe^{2+}	Fe^{3+}#
	Atoms	0.23	0.03	0.13	0.64	0.72	0.70	0.04	0.51	1.02	48.26
Ftc - Map 2 - Point 9		SiO_2	TiO_2	Al_2O_3	Cr_2O_3	Fe_2O_3	FeO	MnO	MgO	TOTAL	
	%Wt	9.76	0.83	5.64	25.46	13.24	25.92	1.79	10.70	93.33	
		Si	Ti	Al	Cr	Fe^{3+}	Fe^{2+}	Mn	Mg	Fe^{3+}/Fe^{2+}	Fe^{3+}#
	Atoms	0.34	0.02	0.23	0.70	0.35	0.75	0.05	0.55	0.46	27.12
Ftc - Map 2 - Point 10		SiO_2	TiO_2	Al_2O_3	Cr_2O_3	Fe_2O_3	FeO	MnO	MgO	TOTAL	
	%Wt	9.62	0.71	5.94	26.22	5.40	23.46	1.41	10.33	83.09	
		Si	Ti	Al	Cr	Fe^{3+}	Fe^{2+}	Mn	Mg	Fe^{3+}/Fe^{2+}	Fe^{3+}#
	Atoms	0.37	0.02	0.27	0.80	0.16	0.75	0.05	0.59	0.21	12.79
Ftc - Map 2 - Point 11		SiO_2	TiO_2	Al_2O_3	Cr_2O_3	Fe_2O_3	FeO	MnO	MgO	TOTAL	
	%Wt	10.11	0.48	6.65	25.62	18.12	26.10	1.94	12.30	101.33	
		Si	Ti	Al	Cr	Fe^{3+}	Fe^{2+}	Mn	Mg	Fe^{3+}/Fe^{2+}	Fe^{3+}#
	Atoms	0.32	0.01	0.25	0.65	0.44	0.70	0.05	0.59	0.62	32.68

Supplementary Table 4 (continued)

Ftc - Map 2 - Point 12		SiO_2	TiO_2	Al_2O_3	Cr_2O_3	Fe_2O_3	FeO	MnO	MgO	TOTAL	
	%Wt	9.48	0.66	6.11	24.58	5.22	23.48	2.17	9.23	80.92	
		Si	Ti	Al	Cr	Fe^{3+}	Fe^{2+}	Mn	Mg	Fe^{3+}/Fe^{2+}	Fe^{3+}#
	Atoms	0.38	0.02	0.29	0.77	0.16	0.78	0.07	0.54	0.20	12.86
Ftc - Map 2 - Point 13		SiO_2	TiO_2	Al_2O_3	Cr_2O_3	Fe_2O_3	FeO	MnO	MgO	TOTAL	
	%Wt	13.86	0.48	4.14	25.94	8.82	32.14	1.46	10.96	97.81	
		Si	Ti	Al	Cr	Fe^{3+}	Fe^{2+}	Mn	Mg	Fe^{3+}/Fe^{2+}	Fe^{3+}#
	Atoms	0.46	0.01	0.16	0.68	0.22	0.89	0.04	0.54	0.25	20.73

Spinel Ftc Rims	SiO_2	TiO_2	Al_2O_3	Cr_2O_3	Fe_2O_3	FeO	MnO	MgO	TOTAL	Fe^{3+}/Fe^{2+}	Fe^{3+}#
Mean	8.98	0.66	9.59	25.29	12.42	25.20	1.83	10.86	94.81	0.43	25.87
Standard deviation (1 σ)	2.24	0.19	9.59	1.13	7.42	4.41	0.37	1.89	6.84	0.26	12.76

Chlorite Chl1

Chlorite - Point 1	***SiO_2***	***TiO_2***	***Al_2O_3***	***Cr_2O_3***	***$(FeO)_T$***	***MnO***	***MgO***	***NiO***	***CaO***	***Na_2O***	***K_2O***	***CoO***	***CuO***	***TOTAL***
	30.37	0.00	19.89	0.02	11.50	0.02	28.04	0.15	0.12	0.00	0.00	0.00	0.08	90.15
	Si	***Ti***	***Al***	***Cr***	***Fe^{2+}***	***Fe^{3+}***	***Mn***	***Mg***	***Ni***	***Ca***	***Na***	***K***	***Cu***	
	5.759	0.000	4.445	0.003	1.824	0.000	0.003	7.924	0.023	0.024	0.000	0.000	0.0115	
	$^{[4]}(Si_{5.759}\ Al_{2.241})\ ^{[6]}(Mg_{7.924}\ Fe^{2+}_{1.824}\ Al_{2.204}\ Cu_{0.0115}\ Ni_{0.023}\ Cr_{0.003})\ ^{[12]}(Ca_{0.024})\ O_{20}\ (OH)_{16}$													
Chlorite - Point 2	***SiO_2***	***TiO_2***	***Al_2O_3***	***Cr_2O_3***	***$(FeO)_T$***	***MnO***	***MgO***	***NiO***	***CaO***	***Na_2O***	***K_2O***	***CoO***	***CuO***	***TOTAL***
	29.27	0.00	20.37	0.03	12.69	0.01	25.31	0.15	0.16	0.02	0.01	0.00	0.00	87.94
	Si	***Ti***	***Al***	***Cr***	***Fe^{2+}***	***Fe^{3+}***	***Mn***	***Mg***	***Ni***	***Ca***	***Na***	***K***	***Cu***	
	5.645	0.000	4.630	0.012	1.314	0.733	0.002	7.275	0.023	0.033	0.000	0.003	0.012	
	$^{[4]}(Si_{5.645}\ Al_{2.355})\ ^{[6]}(Mg_{7.275}\ Fe^{2+}_{1.314}\ Fe^{3+}_{0.733}\ Al_{2.275}\ Mn_{0.002}\ Ni_{0.0233}\ Cr_{0.012}Cu_{0.0115})\ ^{[12]}(Ca_{0.0331}\ Na_{0.0025})\ O_{20}\ (OH)_{16}$													
Chlorite - Point 3	***SiO_2***	***TiO_2***	***Al_2O_3***	***Cr_2O_3***	***$(FeO)_T$***	***MnO***	***MgO***	***NiO***	***CaO***	***Na_2O***	***K_2O***	***CoO***	***CuO***	***TOTAL***
	30.95	0.02	18.59	0.01	10.65	0.00	24.98	0.10	0.43	0.01	0.00	0.08	0.00	85.74
	Si	***Ti***	***Al***	***Cr***	***Fe^{2+}***	***Fe^{3+}***	***Mn***	***Mg***	***Ni***	***Ca***	***Na***	***K***	***Co***	
	6.030	0.003	4.268	0.002	0.892	0.843	0.000	7.254	0.016	0.090	0.004	0.000	0.013	
	$^{[4]}(Si_{6.030}\ Al_{1.970})\ ^{[6]}(Mg_{7.254}\ Fe^{2+}_{0.892}\ Fe^{3+}_{0.843}\ Al_{2.298}\ Co_{0.0125}\ Ni_{0.0157}\ Cr_{0.002}\ Ti_{0.0029})\ ^{[12]}(Ca_{0.0898}\ Na_{0.0038})\ O_{20}\ (OH)_{16}$													

Chlorite Chl1	***SiO_2***	***TiO_2***	***Al_2O_3***	***Cr_2O_3***	***$(FeO)_T$***	***MnO***	***MgO***	***NiO***	***CaO***	***Na_2O***	***K_2O***	***CoO***	***CuO***	***TOTAL***
Mean	*30.20*	*0.01*	*19.62*	*0.02*	*11.62*	*0.01*	*26.11*	*0.13*	*0.23*	*0.01*	*0.00*	*0.03*	*0.03*	*87.94*
Standard deviation (1 σ)	*0.85*	*0.01*	*0.92*	*0.01*	*1.03*	*0.01*	*1.68*	*0.03*	*0.17*	*0.01*	*0.01*	*0.04*	*0.05*	*2.21*

Supplementary Table 5. Microprobe analyses (%Wt), average compositions measured and structural formulas calculated for chlorite rims found around the pseudomorphs of plagioclase (Chl1). To calculate the formulas, we considered as constrain the tetrahedral occupancy (8 atoms), the octahedral occupancy (12 atoms), and the number of total negative charges (56). In order to evaluate the iron oxidation state and its distribution in the tetrahedral and octahedral sheets, the tetrahedral occupancy was fixed at 8. Analyses were performed in punctual mode. $(FeO)_T$ corresponds to total ferric and ferrous iron. Green and red colours correspond to ferrous iron in octahedral coordination and ferric iron in octahedral coordination, respectively. [4] corresponds to the atoms in tetrahedral coordination, [6] corresponds to the atoms in octahedral coordination and [12] corresponds to the atoms in the interlayers.

	**Ferric *vs* ferrous iron and its coordination**	**Tetrahedral occupancy**	**Octahedral occupancy**	**Total negative charges**	**Comments**
Chlorite 1 Point 1	Ferrous iron in octahedral coordination.	8	11.99	56	Very close to the ideal formula of a trioctahedral chlorite in agreement also with the very low content of alkaline cations.
Chlorite 1 Point 2	Ferric and ferrous iron in octahedral coordination.	8	11.32	56	The octahedral occupancy is slightly low; however octahedral vacancies in chlorite are common, mostly when Al^{3+} for Mg^{2+} heterovalent substitutions occur
Chlorite 1 Point 3	Ferric and ferrous iron in octahedral coordination.	8	11.32	56	Same comments as for Chl1 - point 2.
Chlorite 2 Point 1	Ferric and ferrous iron in octahedral coordination.	8	11.77	56	Same comments as for Chl1 - point 1.<br>The Si/Al ratio is closer to the ideal value of 3.
Chlorite 2 Point 2	Ferric and ferrous iron in octahedral coordination.	8	11.76	56	Same comments as for Chl2 - point 1.
Chlorite 2 Point 3	Ferric and ferrous iron in octahedral coordination.	8	11.45	56	Same comments as for Chl2 - point 1 and Chl2 - point 2. The octahedral occupancy and total negative charge are closer to the ideal formula.

Supplementary Table 6. Detailed comments on the chlorite formula and the Fe^{2+} and Fe^{3+} site occupancy, calculated from Supplementary Table 5 and 8. Colours code is the same than in Supplementary Tables 5, 8 and 9.

Hydroandradite Hadr-1

		SiO_2	TiO_2	Al_2O_3	Cr_2O_3	$(FeO)_T$	MnO	MgO	NiO	CaO	Na_2O	K_2O	TOTAL
Hadr-1 Garnet 1	rim	35.64	0.52	3.65	1.76	18.58	0.23	4.72	1.57	30.30	0.00	0.00	96.97
	rim	34.99	0.68	4.67	1.55	14.11	0.41	2.73	0.00	33.41	0.00	0.14	92.67
	rim	32.81	0.91	1.98	1.60	14.07	0.47	0.70	0.00	35.22	0.00	0.14	87.88
	rim	35.20	0.22	3.17	2.37	20.95	0.64	2.26	0.00	33.16	0.12	0.14	98.23
	rim	37.36	0.52	3.15	1.57	20.92	0.23	0.71	0.00	32.31	0.12	0.14	97.04
	rim	34.11	0.98	2.44	1.47	17.56	0.23	3.61	0.00	32.88	0.00	0.00	93.29
	rim	35.20	0.59	3.85	2.71	19.67	0.47	1.44	0.00	27.58	0.00	0.21	91.73
	rim	33.95	0.66	2.48	1.44	21.88	0.10	0.56	0.00	33.25	0.01	0.00	94.33
	rim	36.09	0.83	2.96	1.21	20.93	0.07	3.22	0.00	28.11	0.04	0.00	93.45
Mean rim		35.04	0.66	3.15	1.74	18.74	0.32	2.22	0.17	31.80	0.03	0.08	93.95
Standard deviation (1 σ)		1.32	0.23	0.82	0.48	2.96	0.19	1.48	0.52	2.58	0.05	0.08	3.19
Hadr-1 Garnet 1	core	35.95	1.19	3.71	1.28	20.96	0.29	0.49	0.00	33.18	0.24	0.00	97.29
	core	33.79	1.33	2.46	1.14	23.17	0.46	0.79	0.00	31.59	0.24	0.07	95.05
	core	37.58	1.13	3.54	0.93	14.09	0.18	2.94	0.00	33.97	0.23	0.00	94.60
	core	33.18	2.37	2.39	1.56	21.03	0.07	0.23	0.14	33.40	0.02	0.00	94.40
	core	33.26	1.64	2.64	0.80	23.05	0.04	0.24	0.00	33.51	0.01	0.00	95.21
	core	33.30	1.62	2.57	0.93	22.80	0.06	0.72	0.05	32.62	0.00	0.01	94.67
	core	32.91	1.14	2.32	1.52	22.38	0.08	0.37	0.03	33.57	0.04	0.00	94.34
Mean core		34.28	1.49	2.81	1.16	21.07	0.17	0.83	0.03	33.12	0.11	0.01	95.08
Standard deviation (1 σ)		1.78	0.44	0.57	0.30	3.21	0.16	0.96	0.05	0.79	0.12	0.03	1.03
Hadr-1 Garnet 2	rim	35.20	0.59	3.85	2.71	19.67	0.47	1.44	0.00	27.58	0.00	0.21	91.73
	rim	34.81	0.45	2.78	1.40	17.35	0.58	0.65	1.44	32.58	0.36	0.00	92.40
	rim	35.83	0.60	2.10	0.90	19.75	0.21	0.71	0.00	34.65	0.12	0.07	94.93
	rim	31.93	0.60	2.84	1.42	12.90	0.59	0.51	0.00	31.96	0.34	0.14	83.23
	rim	33.52	0.67	2.53	2.29	19.64	0.53	1.05	0.00	35.03	0.48	0.00	95.74
	rim	33.33	0.59	1.87	1.43	25.39	0.53	1.20	0.00	31.22	0.00	0.14	95.70
	rim	34.77	0.75	3.13	1.97	17.46	0.27	0.39	0.00	36.30	0.12	0.07	95.22
	rim	32.19	0.89	1.87	2.26	21.92	0.21	0.93	0.00	31.21	0.12	0.07	91.68
	rim	35.26	0.53	2.92	2.05	23.09	0.07	0.64	0.08	32.48	0.00	0.00	97.13
	rim	34.58	0.63	2.60	1.37	23.16	0.09	0.44	0.00	33.47	0.02	0.00	96.36
	rim	34.23	0.73	2.72	1.52	23.26	0.13	0.29	0.02	33.95	0.00	0.02	96.85
	rim	34.23	0.63	2.58	1.32	21.95	0.07	0.56	0.09	33.56	0.02	0.01	95.03
	rim	34.96	0.59	2.68	1.85	23.18	0.10	0.33	0.05	33.13	0.00	0.02	96.88
Mean rim		34.22	0.64	2.65	1.73	20.67	0.30	0.70	0.13	32.86	0.12	0.06	94.07
Standard deviation (1 σ)		1.18	0.11	0.53	0.51	3.36	0.21	0.35	0.39	2.16	0.16	0.07	3.76

Supplementary Table 7. Microprobe analyses (%Wt) and average compositions measured on the rims and the cores of two type 1-hydroandradites (Hadr-1) in the Casale serpentinites. Both punctual analyses and microprobe mapping were performed on Garnet 1. Only stage map mode analyses were acquired on Garnet 2. $(FeO)_T$ corresponds to total ferric and ferrous iron. The cores of Hadr-1 display higher TiO_2 content compared to the rims. Hadr-1 contains ~1.6-1.7%Wt Cr_2O_3. Table continues on following page.

Supplementary Table 7 (continued)

		SiO_2	TiO_2	Al_2O_3	Cr_2O_3	$(FeO)_T$	MnO	MgO	NiO	CaO	Na_2O	K_2O	TOTAL
Hadr-1 Garnet 2	core	35.42	1.72	2.10	2.08	18.62	0.59	1.74	0.00	34.60	0.00	0.00	96.87
	core	33.54	1.93	2.65	2.06	18.45	0.58	0.20	0.00	36.11	0.12	0.13	95.77
	core	34.29	1.86	3.04	2.00	20.83	0.48	0.39	0.00	34.43	0.12	0.13	97.57
	core	33.89	1.03	2.15	1.82	24.21	0.37	1.79	0.00	31.97	0.24	0.00	97.48
	core	34.06	1.12	4.06	1.80	18.63	0.32	0.97	0.00	34.86	0.12	0.00	95.92
	core	34.72	1.49	2.37	2.02	18.55	0.32	0.46	0.00	34.01	0.12	0.14	94.20
	core	32.64	1.12	1.59	2.07	19.71	0.53	0.46	0.00	34.58	0.12	0.13	92.94
	core	35.77	1.79	1.85	1.95	20.84	0.64	0.26	0.00	35.01	0.24	0.20	98.55
Mean core		34.29	1.51	2.47	1.97	19.98	0.48	0.78	0.00	34.45	0.13	0.09	96.16
Standard deviation (1 σ)		1.01	0.37	0.78	0.11	1.98	0.13	0.65	0.00	1.17	0.08	0.08	1.87

HYDRO-ANDRADITE Hadr-1	SiO_2	TiO_2	Al_2O_3	Cr_2O_3	$(FeO)_T$	MnO	MgO	NiO	CaO	Na_2O	K_2O	TOTAL
Mean rims	34.55	0.64	2.86	1.74	19.88	0.30	1.32	0.15	32.42	0.08	0.07	94.02
Standard deviation (1 σ)	1.27	0.16	0.69	0.48	3.27	0.20	1.22	0.44	2.34	0.14	0.07	3.46
Mean cores	34.29	1.50	2.63	1.60	20.49	0.33	0.80	0.01	33.83	0.12	0.05	95.66
Standard deviation (1 σ)	1.37	0.39	0.69	0.47	2.59	0.21	0.78	0.04	1.19	0.10	0.07	1.58

Chlorite Chl2

	SiO_2	TiO_2	Al_2O_3	Cr_2O_3	$(FeO)_T$	MnO	MgO	NiO	CaO	Na_2O	K_2O	CoO	CuO	TOTAL
Chlorite - Point 1	30.36	0.02	17.11	0.01	17.72	0.00	25.59	0.22	0.15	0.00	0.01	0.00	0.08	87.21
	Si	Ti	Al	Cr	Fe^{2+}	Fe^{3+}	Mn	Mg	Ni	Ca	Na	K	Cu	
	5.978	0.003	3.970	0.002	1.799	0.461	0.000	7.510	0.035	0.032	0.000	0.003	0.012	
$^{[4]}(Si_{5.978}\ Al_{2.022})\ ^{[6]}(Mg_{7.510}\ Fe^{2+}{}_{1.799}\ Fe^{3+}{}_{0.461}\ Al_{1.949}\ Cu_{0.0119}\ Ni_{0.0349}\ Cr_{0.002}Ti_{0.003})\ ^{[12]}(Ca_{0.0316}\ K_{0.0025})\ O_{20}\ (OH)_{16}$														
	SiO_2	TiO_2	Al_2O_3	Cr_2O_3	$(FeO)_T$	MnO	MgO	NiO	CaO	Na_2O	K_2O	CoO	CuO	TOTAL
Chlorite - Point 2	29.81	0.00	17.48	0.01	14.58	0.00	23.49	0.06	0.17	0.02	0.02	0.00	0.02	85.63
	Si	Ti	Al	Cr	Fe^{2+}	Fe^{3+}	Mn	Mg	Ni	Ca	Na	K	Cu	
	6.029	0.000	4.167	0.002	2.306	0.160	0.000	7.081	0.010	0.037	0.008	0.005	0.006	
$^{[4]}(Si_{6.029}\ Al_{1.971})\ ^{[6]}(Mg_{7.081}\ Fe^{2+}{}_{2.306}\ Fe^{3+}{}_{0.160}\ Al_{2.197}\ Ni_{0.0098}\ Cr_{0.002}Cu_{0.0061})\ ^{[12]}(Ca_{0.0368}\ Na_{0.0078}K_{0,0052})\ O_{20}\ (OH)_{16}$														
	SiO_2	TiO_2	Al_2O_3	Cr_2O_3	$(FeO)_T$	MnO	MgO	NiO	CaO	Na_2O	K_2O	CoO	CuO	TOTAL
Chlorite - Point 3	29.36	0.00	19.36	0.01	14.85	0.00	23.53	0.10	0.10	0.03	0.00	0.04	0.00	87.35
	Si	Ti	Al	Cr	Fe^{2+}	Fe^{3+}	Mn	Mg	Ni	Ca	Na	K	Co	
	5.721	0.000	4.446	0.002	1.249	1.171	0.000	6.834	0.016	0.021	0.011	0.000	0.006	
$^{[4]}(Si_{5.721}\ Al_{2.279})\ ^{[6]}(Mg_{6.834}\ Fe^{2+}{}_{1.249}\ Fe^{3+}{}_{1.171}\ Al_{2.167}\ Co_{0.0063}\ Ni_{0.0157}\ Cr_{0.002}\ )\ ^{[12]}(Ca_{0.0209}\ Na_{0.0113})\ O_{20}\ (OH)_{16}$														

Chlorite Chl2	*SiO_2*	*TiO_2*	*Al_2O_3*	*Cr_2O_3*	*$(FeO)_T$*	*MnO*	*MgO*	*NiO*	*CaO*	*Na_2O*	*K_2O*	*CoO*	*CuO*	*TOTAL*
Mean	*29.84*	*0.01*	*17.98*	*0.01*	*15.72*	*0.00*	*24.20*	*0.13*	*0.14*	*0.02*	*0.01*	*0.01*	*0.03*	*86.73*
Standard deviation (1 σ)	*0.50*	*0.01*	*1.21*	*0.00*	*1.74*	*0.00*	*1.20*	*0.08*	*0.04*	*0.02*	*0.01*	*0.02*	*0.04*	*0.96*

Supplementary Table 8. Microprobe analyses (%Wt), average compositions measured and structural formulas calculated for the chlorite from the Chl2+Srp+Hadr-2 assemblages substituting the plagioclase. To calculate the formulas, we considered as constrain the tetrahedral occupancy (8 cations), the octahedral occupancy (12 cations), and the number of total negative charges (56). In order to evaluate the iron oxidation state and its distribution in the tetrahedral and octahedral sheets, the tetrahedral occupancy was fixed at 8. Analyses were performed in punctual mode. $(FeO)_T$ corresponds to total ferric and ferrous iron. Green and red colours correspond to ferrous iron in octahedral coordination and ferric iron in octahedral coordination, respectively. [4] corresponds to the atoms in tetrahedral coordination, [6] corresponds to the atoms in octahedral coordination and [12] corresponds to the atoms in the interlayers.

Hydroandradite Hadr-2

	SiO_2	TiO_2	Al_2O_3	Cr_2O_3	$(FeO)_T$	MnO	MgO	NiO	CaO	Na_2O	K_2O	TOTAL
Hadr-2 Map1	36.10	0.30	4.14	0.33	22.02	0.43	4.02	0.00	31.20	0.35	0.21	99.09
	36.22	0.38	6.94	0.40	16.38	0.32	4.38	0.00	32.69	0.22	0.27	98.21
	30.14	0.15	2.47	0.17	12.96	0.48	10.91	0.00	20.07	0.33	0.14	77.83
	35.90	0.22	4.22	0.28	19.84	0.27	9.03	0.00	22.97	0.57	0.00	93.29
	34.80	0.59	4.84	0.33	23.10	0.37	3.73	1.44	27.70	0.00	0.00	96.90
	33.70	0.23	3.03	0.45	17.50	0.59	2.81	0.00	32.10	0.12	0.14	90.66
	34.68	0.04	3.48	0.01	23.62	0.00	1.54	0.13	31.29	0.04	0.01	94.84
Mean	34.51	0.27	4.16	0.28	19.35	0.35	5.20	0.22	28.29	0.23	0.11	92.97
Standard deviation (1 σ)	2.13	0.18	1.46	0.15	3.93	0.19	3.43	0.54	4.96	0.20	0.11	7.29
Hadr-2 Map2	34.95	0.37	4.47	0.33	22.05	0.27	0.13	0.00	32.56	0.00	0.07	95.21
	36.83	0.37	6.17	0.34	19.76	0.27	1.61	0.00	33.25	0.23	0.00	98.83
	32.87	0.08	6.38	0.40	16.37	0.22	1.39	0.00	34.67	0.00	0.14	92.52
	31.82	0.23	4.46	0.28	15.16	0.38	1.35	0.00	32.79	0.23	0.20	86.89
	31.57	0.23	4.08	0.11	18.61	0.21	0.71	0.00	36.42	0.23	0.00	92.19
	32.41	0.15	3.52	0.33	20.90	0.48	4.38	0.00	25.09	0.35	0.07	87.68
	33.22	0.15	6.02	0.11	23.18	0.37	3.25	0.00	29.85	0.35	0.14	96.65
	29.99	0.38	4.06	0.29	12.92	0.32	2.71	0.00	32.38	0.34	0.20	83.59
	29.56	0.15	3.45	0.38	27.47	0.47	4.18	1.43	31.90	0.25	0.14	99.37
	29.90	0.30	3.25	0.11	24.32	0.26	2.78	0.00	27.63	0.12	0.07	88.74
	32.04	0.07	2.98	0.17	23.04	0.32	2.13	1.44	30.04	0.36	0.07	92.65
	30.75	0.15	5.84	0.44	23.08	0.42	3.95	1.44	27.70	0.12	0.21	94.10
	33.28	0.08	7.71	0.40	12.92	0.32	3.37	0.00	27.31	0.22	0.00	85.61
	35.44	0.60	4.54	0.50	19.72	0.37	3.37	1.44	27.77	0.12	0.14	94.00
	29.47	0.15	5.32	0.56	15.20	0.48	3.83	0.00	27.88	0.23	0.00	83.12
Mean	32.27	0.23	4.82	0.32	19.65	0.34	2.61	0.38	30.48	0.21	0.10	91.41
Standard deviation (1 σ)	2.23	0.15	1.37	0.14	4.38	0.09	1.32	0.66	3.22	0.12	0.08	5.23

Supplementary Table 9. Microprobe analyses (%Wt) and average compositions measured on type 2-hydroandradites (Hadr-2) in the Casale serpentinites. The analyses reported here were retrieved from maps performed on four Hadr-2 crystals. No chemical zonation was observed and thus there is no core/rim distinction for the reported analyses. $(FeO)_T$ corresponds to total ferric and ferrous iron. Contrarily to Hadr-1, Cr_2O_3 content is low (~0.4%Wt). Table continues on following pages.

Supplementary Table 9 (continued)

	SiO_2	TiO_2	Al_2O_3	Cr_2O_3	$(FeO)_T$	MnO	MgO	NiO	CaO	Na_2O	K_2O	TOTAL
Hadr-2 Map3	32.44	0.38	2.56	0.49	16.52	0.41	1.33	0.00	34.26	0.13	0.15	88.66
	31.90	0.52	5.52	0.55	19.00	0.41	3.79	0.00	30.80	0.25	0.00	92.74
	33.50	0.15	6.03	0.85	22.70	0.29	6.10	0.00	29.40	0.00	0.00	99.02
	38.20	0.38	2.84	0.31	15.30	0.35	14.50	0.00	23.70	0.00	0.08	95.65
	34.00	0.30	6.25	0.48	25.20	0.17	7.68	0.00	24.40	0.13	0.00	98.60
	35.00	0.22	2.37	0.42	22.70	0.23	4.99	0.00	23.70	0.00	0.00	89.63
	35.30	0.22	3.89	0.83	27.50	0.46	5.70	0.00	23.60	0.26	0.07	97.84
Mean	34.33	0.31	4.21	0.56	21.27	0.33	6.30	0.00	27.12	0.11	0.04	94.59
Standard deviation (1 σ)	2.11	0.13	1.70	0.20	4.50	0.10	4.13	0.00	4.34	0.12	0.06	4.29
Hadr-2 Map4	37.62	0.20	5.40	0.42	21.43	0.23	3.55	1.56	27.05	0.00	0.00	97.47
	33.87	0.20	4.22	0.54	27.68	0.57	1.01	0.00	30.93	0.27	0.07	99.37
	36.50	0.34	6.30	0.79	16.50	0.58	2.37	0.00	31.40	0.25	0.30	95.34
	33.20	0.34	3.77	0.67	16.50	0.58	4.12	0.00	31.20	0.25	0.00	90.64
	34.20	0.27	7.74	0.60	22.70	0.12	4.09	0.00	27.10	0.00	0.08	96.89
	37.00	0.21	5.52	0.79	21.60	0.17	5.28	0.00	27.50	0.00	0.08	98.14
	36.00	0.48	5.63	0.48	21.60	0.35	2.60	0.00	28.10	0.00	0.15	95.39
	34.70	0.28	5.55	0.31	17.90	0.41	1.94	0.00	29.10	0.13	0.00	90.30
	35.70	0.27	4.01	0.36	19.00	0.23	0.35	1.56	32.40	0.00	0.07	93.97
	34.90	0.14	4.41	0.37	19.10	0.23	0.42	0.00	32.80	0.13	0.00	92.49
	30.60	0.21	5.41	0.55	16.60	0.35	1.05	0.00	34.20	0.51	0.15	89.62
	34.00	0.41	4.58	0.24	22.80	0.29	0.43	0.00	33.50	0.13	0.07	96.45
	34.30	0.34	4.15	0.67	22.80	0.29	0.21	0.00	34.80	0.13	0.00	97.69
	32.20	0.00	5.11	0.43	12.80	0.47	1.18	1.57	29.60	0.00	0.15	83.51
	34.90	0.27	5.49	0.67	19.00	0.29	2.05	0.00	33.30	0.39	0.00	96.36
	32.90	0.14	6.04	0.37	17.80	0.35	2.99	0.00	31.70	0.13	0.00	92.41
	33.00	0.41	5.54	0.42	21.40	0.40	2.57	0.00	29.00	0.13	0.07	92.95
	35.80	0.27	3.80	0.00	25.20	0.40	2.80	1.56	29.50	0.13	0.07	99.54
	32.90	0.20	4.52	0.42	23.90	0.17	1.94	0.00	32.00	0.00	0.07	96.13
	34.50	0.14	6.29	0.42	22.80	0.12	0.85	0.00	33.50	0.00	0.07	98.69
	34.20	0.34	5.25	0.24	22.60	0.58	2.29	1.56	31.80	0.00	0.00	98.86
	34.90	0.28	4.25	0.43	15.30	0.35	2.00	0.00	33.50	0.13	0.07	91.21
	34.00	0.14	5.71	0.37	16.50	0.47	1.19	0.00	36.70	0.13	0.07	95.27
	34.00	0.27	6.04	0.42	21.50	0.52	0.92	0.00	31.70	0.00	0.15	95.53
	32.20	0.34	6.09	0.24	17.60	0.29	1.50	1.56	33.50	0.00	0.30	93.62
	33.90	0.28	5.16	0.37	17.80	0.06	2.37	0.00	32.80	0.00	0.00	92.73
	35.00	0.21	4.06	0.37	17.80	0.23	4.66	0.00	31.20	0.13	0.23	93.88
	35.10	0.27	4.91	0.49	20.30	0.29	2.81	0.00	31.70	0.13	0.15	96.15
	32.10	0.20	4.09	0.30	20.40	0.23	13.30	0.00	22.20	0.25	0.00	93.08
	32.50	0.41	3.25	0.18	20.40	0.17	8.19	0.00	29.90	0.00	0.15	95.16
	33.30	0.28	4.31	0.18	15.30	0.35	2.92	0.00	33.00	0.38	0.15	90.17

Supplementary Table 9 (continued)

	SiO_2	TiO_2	Al_2O_3	Cr_2O_3	$(FeO)_T$	MnO	MgO	NiO	CaO	Na_2O	K_2O	TOTAL
Hadr-2 Map4	34.50	0.21	7.68	0.49	17.70	0.46	3.84	1.57	29.10	0.13	0.15	95.82
	34.50	0.56	7.82	0.19	12.80	0.59	1.08	0.00	32.90	0.00	0.07	90.50
	31.50	0.75	4.92	0.24	21.50	0.69	0.72	1.56	31.40	0.13	0.07	93.48
	31.50	0.55	5.27	0.43	15.30	0.17	1.46	0.00	34.50	0.13	0.00	89.31
	36.90	0.42	6.00	0.37	14.10	0.35	0.61	0.00	37.40	0.12	0.30	96.58
	31.50	0.55	4.56	0.37	17.80	0.52	1.40	0.00	33.60	0.13	0.07	90.50
	31.70	0.41	5.72	0.12	26.20	0.11	0.95	1.55	34.90	0.00	0.00	101.66
Mean	***34.00***	***0.30***	***5.23***	***0.40***	***19.47***	***0.34***	***2.47***	***0.37***	***31.59***	***0.11***	***0.09***	***94.39***
Standard deviation (1 σ)	***1.69***	***0.14***	***1.08***	***0.18***	***3.57***	***0.16***	***2.42***	***0.67***	***2.92***	***0.12***	***0.09***	***3.60***

HYDRO-ANDRADITE HADR-2	***SiO_2***	***TiO_2***	***Al_2O_3***	***Cr_2O_3***	***$(FeO)_T$***	***MnO***	***MgO***	***NiO***	***CaO***	***Na_2O***	***K_2O***	***TOTAL***
Mean	*33.70*	*0.28*	*4.92*	*0.39*	*19.69*	*0.34*	*3.19*	*0.32*	*30.53*	*0.15*	*0.09*	*93.60*
Standard deviation (1 σ)	*2.02*	*0.15*	*1.30*	*0.18*	*3.84*	*0.14*	*2.85*	*0.62*	*3.65*	*0.14*	*0.08*	*4.60*

Saponite

	SiO_2	TiO_2	Al_2O_3	Cr_2O_3	$(FeO)_T$		MnO	MgO	NiO	CaO	Na_2O	K_2O	TOTAL
Saponite - Point 1	53.61	0.02	7.55	0.01	3.90		0.00	26.42	0.41	0.43	0.22	0.04	92.61
	Si	Ti	Al	Cr	Fe^{2+}	Fe^{3+}	Mn	Mg	Ni	Ca	Na	K	
	6.861	0.002	1.138	0.001	0.000	0.376	0.000	5.039	0.042	0.059	0.06	0.007	
$^{[4]}(Si_{6.861}\ Al_{1.139})\ ^{[6]}(Mg_{5.040}\ Fe^{3+}_{0.376}\ Ti_{0.0019}\ Cr_{0.001}\ Ni_{0.0422})\ ^{[12]}(Ca_{0.059}\ Na_{0.0546}\ K_{0.007})\ O_{20}\ (OH)_4$													
	SiO_2	TiO_2	Al_2O_3	Cr_2O_3	$(FeO)_T$		MnO	MgO	NiO	CaO	Na_2O	K_2O	TOTAL
Saponite - Point 2	43.67	0.01	6.56	0.02	3.88		0.01	27.56	0.50	0.74	0.28	0.06	83.29
	Si	Ti	Al	Cr	Fe^{2+}	Fe^{3+}	Mn	Mg	Ni	Ca	Na	K	
	6.617	0.001	1.171	0.002	0.232	0.212	0.001	6.224	0.061	0.120	0.082	0.012	
$^{[4]}(Si_{6.617}\ Al_{1.171}Fe^{3+}_{0.212})\ ^{[6]}(Mg_{6.224}\ Fe^{2+}_{0.232}\ Ti_{0.0011}\ Mn_{0.001}Cr_{0.002}\ Ni_{0.0609})\ ^{[12]}(Ca_{0.120}\ Na_{0.0823}\ K_{0.0116})\ O_{20}\ (OH)_4$													
	SiO_2	TiO_2	Al_2O_3	Cr_2O_3	$(FeO)_T$		MnO	MgO	NiO	CaO	Na_2O	K_2O	TOTAL
Saponite - Point 3	49.72	0.00	7.36	0.00	3.82		0.01	28.45	0.28	0.69	0.27	0.04	90.66
	Si	Ti	Al	Cr	Fe^{2+}	Fe^{3+}	Mn	Mg	Ni	Ca	Na	K	
	6.812	0.000	1.188	0.000	0.000	0.394	0.001	5.809	0.031	0.101	0.072	0.007	
$^{[4]}(Si_{6.812}\ Al_{1.188})\ ^{[6]}(Mg_{5.809}\ Fe^{3+}_{0.394}\ Mn_{0.001}Ni_{0.0309})\ ^{[12]}(Ca_{0.101}\ Na_{0.0717}\ K_{0.007})\ O_{20}\ (OH)_4$													
	SiO_2	TiO_2	Al_2O_3	Cr_2O_3	$(FeO)_T$		MnO	MgO	NiO	CaO	Na_2O	K_2O	TOTAL
Saponite - Point 4	51.63	0.02	6.90	0.00	3.72		0.00	26.02	0.37	0.80	0.20	0.05	89.71
	Si	Ti	Al	Cr	Fe^{2+}	Fe^{3+}	Mn	Mg	Ni	Ca	Na	K	
	6.911	0.002	1.089	0.000	0.000	0.375	0.000	5.192	0.040	0.115	0.052	0.009	
$^{[4]}(Si_{6,911}\ Al_{1.089})\ ^{[6]}(Mg_{5.192}\ Fe^{3+}_{0.375}\ Ti_{0.002}\ Ni_{0.0398})\ ^{[12]}(Ca_{0.115}\ Na_{0.0519}\ K_{0.0085})\ O_{20}\ (OH)_4$													
	SiO_2	TiO_2	Al_2O_3	Cr_2O_3	$(FeO)_T$		MnO	MgO	NiO	CaO	Na_2O	K_2O	TOTAL
Saponite - Point 5	44.12	0.01	5.58	0.02	3.51		0.00	24.98	0.45	0.93	0.26	0.05	79.90
	Si	Ti	Al	Cr	Fe^{2+}	Fe^{3+}	Mn	Mg	Ni	Ca	Na	K	
	6.946	0.001	1.035	0.000	0.398	0.019	0.000	5.862	0.057	0.157	0.079	0.010	
$^{[4]}(Si_{6.946}\ Al_{1.035}Fe^{3+}_{0.019})\ ^{[6]}(Mg_{5.862}\ Fe^{2+}_{0.398}\ Ti_{0.0012}\ Cr_{0.002}\ Ni_{0.057})\ ^{[12]}(Ca_{0.157}\ Na_{0.0794}\ K_{0.0100})\ O_{20}\ (OH)_4$													
	SiO_2	TiO_2	Al_2O_3	Cr_2O_3	$(FeO)_T$		MnO	MgO	NiO	CaO	Na_2O	K_2O	TOTAL
Saponite - Point 6	48.89	0.01	5.91	0.00	4.35		0.02	24.81	0.36	2.65	0.25	0.04	87.28
	Si	Ti	Al	Cr	Fe^{2+}	Fe^{3+}	Mn	Mg	Ni	Ca	Na	K	
	7.002	0.001	0.998	0.000	0.000	0.469	0.002	5.297	0.041	0.406	0.069	0.007	
$^{[4]}(Si_{7.002}\ Al_{0,998})\ ^{[6]}(Mg_{5.296}\ Fe^{3+}_{0.469}\ Ti_{0.0011}\ Mn_{0.002}\ Ni_{0.0415})\ ^{[12]}(Ca_{0.407}\ Na_{0.0694}\ K_{0.007})\ O_{20}\ (OH)_4$													
	SiO_2	TiO_2	Al_2O_3	Cr_2O_3	$(FeO)_T$		MnO	MgO	NiO	CaO	Na_2O	K_2O	TOTAL
Saponite - Point 7	45.57	0.05	5.91	0.01	6.11		0.01	23.73	0.45	5.50	0.20	0.04	87.57
	Si	Ti	Al	Cr	Fe^{2+}	Fe^{3+}	Mn	Mg	Ni	Ca	Na	K	
	6.867	0.006	1.050	0.001	0.578	0.084	0.001	5.330	0.055	0.888	0.058	0.008	
$^{[4]}(Si_{6.867}\ Al_{1.050}Fe^{3+}_{0.084})\ ^{[6]}(Mg_{5.330}\ Fe^{2+}_{0.578}Fe^{3+}_{0.0295}\ Ti_{0.0057}\ Mn_{0.001}Cr_{0.001}Ni_{0.0546})\ ^{[12]}(Ca_{0.888}\ Na_{0.0584}\ K_{0.0077})\ O_{20}\ (OH)_4$													

Supplementary Table 10. Microprobe analyses (%Wt) and average compositions measured on saponite in the Casale serpentinites and their calculated formulas These analyses show a $(FeO)_T$ content up to 6%Wt. To calculate the formulas, we considered as constrain the tetrahedral occupancy (8 cations), the octahedral occupancy (6 cations), and the number of total negative charges (44). In order to evaluate the iron oxidation state and its distribution in the tetrahedral and octahedral sheets, we fixed the tetrahedral occupancy at 8. Analyses were performed in punctual mode. $(FeO)_T$ corresponds to total ferric and ferrous iron. Red and blue Fe^{3+} correspond to ferric iron in octahedral coordination and tetrahedral coordination, respectively. Green Fe^{2+} corresponds to ferrous iron in octahedral coordination. [4] corresponds to the atoms in tetrahedral coordination, [6] corresponds to the atoms in octahedral coordination and [12] corresponds to the atoms in the interlayers. *Table continues on next page.*

Supplementary Table 10 (continued)

Point 8	SiO_2	TiO_2	Al_2O_3	Cr_2O_3	$(FeO)_T$		MnO	MgO	NiO	CaO	Na_2O	K_2O	TOTAL
	43.92	0.02	5.19	0.03	5.48		0.03	34.90	0.00	0.58	0.04	0.00	90.20
Saponite layer	SiO_2	TiO_2	Al_2O_3	Cr_2O_3	$(FeO)_T$		MnO	MgO	NiO	CaO	Na_2O	K_2O	TOTAL
	2.22	0.02	0.26	0.03	1.27		0.03	1.77	0.00	0.58	0.04	0.00	6.23
	Si	Ti	Al	Cr	Fe^{2+}	Fe^{3+}	Mn	Mg	Ni	Ca	Na	K	
	4.945	0.033	0.689	0.053	0.000	2.366	0.056	5.857	0.000	1.381	0.172	0.000	
	$^{[4]}(Si_{4.945}Al_{0.689}Fe^{3+}_{2.399})\ ^{[6]}(Mg_{5.857}Ti_{0.0034}Mn_{0.056}Cr_{0.053})\ ^{[12]}(Ca_{0.888}\ Na_{0.0584}\ K_{0.0077})\ O_{20}\ (OH)_4$												
Chlorite layer	SiO_2	TiO_2	Al_2O_3	Cr_2O_3	$(FeO)_T$		MnO	MgO	NiO	CaO	Na_2O	K_2O	TOTAL
	41.70	0.02	4.93	0.03	4.07		0.03	33.13	0.00	0.00	0.00	0.00	83.91
	Si	Ti	Al	Cr	Fe^{2+}	Fe^{3+}	Mn	Mg	Ni	Ca	Na	K	
	7.989	0.003	0.011	0.005	0.000	0.587	0.005	9.460	0.000	0.000	0.000	0.000	
	$^{[4]}(Si_{7.989}Al_{0.0113})\ ^{[6]}(Mg_{9.460}Fe^{3+}_{0,587}Al_{1,102}Ti_{0.0029}Mn_{0.005}Cr_{0.004})\ O_{20}\ (OH)_{16}$												
Point 9	SiO_2	TiO_2	Al_2O_3	Cr_2O_3	$(FeO)_T$		MnO	MgO	NiO	CaO	Na_2O	K_2O	TOTAL
	43.06	0.02	5.22	0.01	6.93		0.01	35.77	0.00	5.14	0.02	0.01	96.19
Chlorite layer	Si	Ti	Al	Cr	Fe^{2+}	Fe^{3+}	Mn	Mg	Ni	Ca	Na	K	
	7.832	0.003	1.091	0.004	0.000	0.735	0.005	9.276	0.000	0.111	0.014	0.000	
	$^{[4]}(Si_{7.832}Al_{0.168})\ ^{[6]}(Mg_{9.276}Fe^{3+}_{0.735}Al_{0.923}Ti_{0.0027}Mn_{0.005}Cr_{0.004})\ ^{[12]}(Ca_{0.111}Na_{0.013})\ O_{20}\ (OH)_{16}$												

	Ferric *vs* ferrous iron and its coordination	Tetrahedral occupancy	Octahedral occupancy	Total negative charges	Comments
Point 1	Ferric iron in octahedral coordination.	8	5.46	42.34	Possible presence of a dioctahedral component and vacancies in the octahedral sheet.
Point 2	Ferric iron in tetrahedral coordination and ferrous iron in octahedral coordination.	8	6.52	43.99	It is necessary to hypothesize that part of iron is in tetrahedral coordination as, if not, total negative charge should be in excess. The excess of octahedral occupancy support the hypothesis that part of Fe, Ni, Cr, Mn, and Mg are in the interlayer position, even if, usually, these elements are in octahedral coordination.
Point 3	Ferric iron in octahedral coordination.	8	6.24	43.96	Like in point 3, the excess of octahedral occupancy support that part of Fe, Ni, Cr, Mn, and Mg are in the interlayer position.
Point 4	Ferric iron in octahedral coordination.	8	5.61	42.8	Same comments as for point 1.
Point 5	Ferric iron in tetrahedral coordination and ferrous iron in octahedral coordination.	8	6.32	43.99	Same comments as for point 2.
Point 6	Ferric iron in octahedral coordination.	8	5.81	43.98	Same comments as for points 1 and 5, but with octahedral occupancy and total negative charge closer to ideal formula.
Point 7	Ferric iron in tetrahedral coordination and both ferric and ferrous iron in octahedral coordination.	8	6	44.75	The high negative charge can be explained supposing partial dehydroxylation of the octahedral sheet.
Point 8 Saponite layer	Ferric iron in tetrahedral coordination.	8	6	44	The high content in Mg, the relative low content in Si and Ca, and the low L.O.I. nicely agree with the presence of mixed layer saponite/chlorite. It is possible to calculate the formula of saponite excluding chlorite and vice versa. For chlorite only, the low octahedral occupancy can be explained supposing vacancies in both octahedral sheets; it should be pointed out also that the tetrahedral Al calculated for chlorite after calculating saponite (i.e., from the residual atoms) is very low (commonly it varies between 0.9 and 1.1). It is also possible to use all the elements to calculate a chlorite formula, but with a low octahedral occupancy; moreover Ca and Na must be placed in interlayer with the octahedral sheet.
Point 8 Chlorite layer	Ferric iron in octahedral coordination.	8	11.16	56	
Point 9 Chlorite layer	Ferric iron in octahedral coordination.	8	10.95	55.63	Same comment as point 8

Supplementary Table 11. Detailed comments on the saponite formula and the Fe^{2+} and Fe^{3+} site occupancy, calculated from ***Supplementary Table 10****. Colours code is the same than in Supplementary Tables 5, 8 and 10.*

Hematite

Raw data

		SiO_2	TiO_2	Al_2O_3	Cr_2O_3	$(FeO)_T$	MnO	MgO	NiO	CaO	Na_2O	K_2O	CuO	TOTAL
Hematite Analysis 1	rim	2.09	0.26	0.17	0.05	87.06	0.00	0.33	0.12	0.22	0.00	0.04	0.20	90.55
	rim	2.02	0.27	0.22	0.01	86.52	0.00	0.49	0.07	0.32	0.00	0.04	0.00	89.97
	rim	2.04	0.29	0.21	0.04	86.79	0.00	0.44	0.00	0.24	0.01	0.03	0.07	90.16
	core	1.49	0.06	0.14	0.02	89.20	0.00	0.29	0.01	0.04	0.00	0.00	0.06	91.32
	core	1.34	0.00	0.15	0.05	88.07	0.01	0.16	0.07	0.08	0.07	0.00	0.13	90.13
	core	0.53	0.02	0.06	0.01	90.09	0.00	0.14	0.24	0.03	0.00	0.01	0.09	91.24
Hematite Analysis 2	rim	0.79	0.16	0.17	0.36	88.37	0.02	0.18	0.00	0.35	0.00	0.01	0.00	90.41
	core	0.41	0.02	0.11	0.10	88.93	0.02	0.09	0.00	0.05	0.00	0.02	0.04	89.79
Hematite Analysis 3	rim	3.28	0.26	0.64	0.03	84.25	0.01	1.80	0.19	0.11	0.02	0.04	0.00	90.64

After recalculation Fe^{3+}/Fe^{2+}

		SiO_2	TiO_2	Al_2O_3	Cr_2O_3	Fe_2O_3	MnO	MgO	NiO	CaO	Na_2O	K_2O	CuO	TOTAL
Hematite Analysis 1	rim	2.09	0.26	0.17	0.05	96.75	0.00	0.33	0.12	0.22	0.00	0.04	0.20	100.24
	rim	2.02	0.27	0.22	0.01	96.15	0.00	0.49	0.07	0.32	0.00	0.04	0.00	99.60
	rim	2.04	0.29	0.21	0.04	96.44	0.00	0.44	0.00	0.24	0.01	0.03	0.07	99.81
Mean rim		***2.05***	***0.27***	***0.20***	***0.03***	***96.45***	***0.00***	***0.42***	***0.07***	***0.26***	***0.00***	***0.04***	***0.09***	***99.88***
Standard deviation (1 σ)		***0.04***	***0.02***	***0.03***	***0.02***	***0.30***	***0.00***	***0.08***	***0.06***	***0.05***	***0.01***	***0.01***	***0.10***	***0.33***
Hematite Analysis 1	core	1.49	0.06	0.14	0.02	99.13	0.00	0.29	0.00	0.04	0.00	0.00	0.00	101.17
	core	1.34	0.00	0.15	0.05	97.87	0.01	0.16	0.07	0.08	0.07	0.00	0.13	99.93
	core	0.53	0.02	0.06	0.01	100.12	0.00	0.14	0.24	0.03	0.00	0.01	0.09	101.27
Mean core		***1.12***	***0.03***	***0.12***	***0.03***	***99.04***	***0.00***	***0.20***	***0.11***	***0.05***	***0.02***	***0.00***	***0.08***	***75.30***
Standard deviation (1 σ)		***0.51***	***0.03***	***0.05***	***0.02***	***1.13***	***0.01***	***0.08***	***0.12***	***0.03***	***0.04***	***0.00***	***0.07***	***0.75***
Hematite Analysis 2	rim	0.79	0.16	0.17	0.36	98.20	0.02	0.18	0.00	0.35	0.00	0.01	0.00	100.25
	core	0.41	0.02	0.11	0.10	98.83	0.02	0.09	0.00	0.05	0.00	0.02	0.04	99.69
Hematite Analysis 3	rim	3.28	0.26	0.64	0.03	93.62	0.01	1.80	0.19	0.11	0.02	0.04	0.00	100.02

Hematite	SiO_2	TiO_2	Al_2O_3	Cr_2O_3	Fe_2O_3	MnO	MgO	NiO	CaO	Na_2O	K_2O	CuO	TOTAL
Mean Cores	*0.94*	*0.03*	*0.12*	*0.05*	*98.99*	*0.01*	*0.17*	*0.08*	*0.05*	*0.02*	*0.01*	*0.07*	*100.51*
Standard deviation (1 σ)	*0.55*	*0.02*	*0.04*	*0.04*	*0.93*	*0.01*	*0.09*	*0.11*	*0.02*	*0.03*	*0.01*	*0.06*	*0.82*
Mean Rims	*2.04*	*0.25*	*0.28*	*0.10*	*96.23*	*0.01*	*0.65*	*0.08*	*0.25*	*0.01*	*0.03*	*0.06*	*99.98*
Standard deviation (1 σ)	*0.88*	*0.05*	*0.20*	*0.15*	*1.66*	*0.01*	*0.66*	*0.08*	*0.09*	*0.01*	*0.01*	*0.09*	*0.28*

Supplementary Table 12. Raw microprobe analyses (%Wt) and average compositions measured on hematite from the CCM-HemSap assemblages of the Casale serpentinites (see main text for acronym definition). Analyses displayed here were performed in punctual mode (Hematite 2 and 3) or were retrieved from a map (Hematite 1). $(FeO)_T$ corresponds to total ferric and ferrous iron. Normalized analyses after Fe^{3+}/Fe^{2+} recalculation are also reported.

Hydroandradite Hadr-3

	SiO_2	TiO_2	Al_2O_3	Cr_2O_3	$(FeO)_T$	MnO	MgO	NiO	CaO	Na_2O	K_2O	TOTAL
	34.51	0.15	3.95	0.39	16.31	0.32	2.43	1.44	29.31	0.12	0.14	89.07
	35.82	0.37	4.20	0.22	25.41	0.16	4.53	0.00	25.66	0.00	0.14	96.49
	32.91	0.08	4.49	0.23	15.20	0.54	3.75	0.00	28.61	0.57	0.21	86.57
	37.45	0.75	3.49	0.28	19.79	0.32	6.45	0.00	27.29	0.00	0.07	95.89
	34.59	0.60	4.38	0.17	14.04	0.38	5.60	0.00	27.67	0.00	0.00	87.43
	36.37	0.45	4.44	0.34	16.19	0.32	4.92	2.88	26.88	0.35	0.14	93.26
Hadr-3 Garnet 3	35.31	0.30	3.59	0.11	20.84	0.43	2.22	1.44	31.41	0.24	0.14	96.01
	36.65	0.00	5.64	0.34	17.54	0.32	9.24	0.00	29.86	0.11	0.21	99.92
	37.42	0.38	4.63	0.28	16.36	0.16	5.36	0.00	29.16	0.11	0.14	94.00
	32.36	0.08	6.10	0.45	10.56	0.32	6.28	0.00	23.81	0.11	0.07	80.15
	33.35	0.23	3.65	0.29	10.59	0.32	4.40	0.00	32.11	0.22	0.00	85.17
	38.43	0.22	4.56	0.11	17.33	0.21	4.46	2.88	28.53	0.47	0.14	97.35
	36.21	0.15	6.28	0.33	24.24	0.32	7.16	1.44	23.13	0.35	0.00	99.61
**Mean**	**35.49**	**0.29**	**4.57**	**0.27**	**17.26**	**0.32**	**5.14**	**0.78**	**27.96**	**0.20**	**0.11**	**92.38**
Standard deviation (1 σ)	**1.87**	**0.22**	**0.91**	**0.10**	**4.48**	**0.10**	**1.90**	**1.12**	**2.65**	**0.18**	**0.07**	**6.13**
	38.87	1.51	2.63	0.49	18.92	0.46	4.85	0.00	31.01	0.13	0.00	98.85
	34.94	2.13	2.03	0.43	19.04	0.23	4.26	0.00	29.47	0.13	0.07	92.73
	33.93	1.93	2.43	0.25	16.55	0.41	2.64	0.00	29.78	0.13	0.00	88.05
	34.67	1.26	2.39	0.44	11.53	0.41	2.91	0.00	35.53	0.24	0.15	89.54
	36.91	1.65	1.61	0.49	19.02	0.35	6.13	0.00	28.93	0.25	0.15	95.49
	37.29	2.09	2.00	0.31	12.81	0.12	6.00	0.00	33.37	0.00	0.00	93.99
	35.19	1.67	1.29	0.74	12.81	0.18	5.97	0.00	29.66	0.12	0.22	87.86
	33.98	1.78	3.32	0.30	20.27	0.35	2.95	0.00	31.30	0.13	0.00	94.38
	35.42	2.18	3.34	0.60	23.90	0.35	2.28	0.00	31.03	0.26	0.22	99.58
	36.94	1.99	3.46	0.55	17.74	0.23	3.55	0.00	30.24	0.13	0.15	94.98
Hadr-3 Garnet 4	37.52	1.02	1.61	0.24	22.75	0.29	4.65	0.00	28.74	0.13	0.30	97.24
	36.56	1.29	2.91	0.54	22.64	0.17	2.07	1.56	29.71	0.26	0.15	97.86
	33.43	0.95	2.34	0.42	22.63	0.29	2.52	1.56	29.53	0.26	0.00	93.93
	34.62	0.96	1.15	0.18	18.98	0.06	2.40	0.00	35.12	0.00	0.07	93.56
	34.47	1.65	3.15	0.18	19.03	0.35	1.47	0.00	31.13	0.38	0.22	92.04
	33.94	0.75	1.89	0.18	22.65	0.40	2.15	0.00	32.13	0.13	0.07	94.30
	35.73	1.22	2.18	0.36	23.96	0.35	1.79	0.00	29.78	0.39	0.22	95.98
	38.14	1.03	2.77	0.24	18.80	0.40	2.43	3.12	30.85	0.39	0.30	98.48
	34.38	0.81	1.90	0.36	25.06	0.63	1.38	0.00	31.86	0.13	0.07	96.60
	33.25	2.39	1.89	0.30	18.95	0.52	2.63	0.00	29.58	0.26	0.00	89.78
	34.38	0.81	1.90	0.36	25.06	0.63	1.38	0.00	31.86	0.13	0.07	96.60
**Mean**	**35.46**	**1.48**	**2.29**	**0.38**	**19.67**	**0.34**	**3.16**	**0.30**	**30.98**	**0.19**	**0.12**	**94.37**
Standard deviation (1 σ)	**1.62**	**0.51**	**0.67**	**0.15**	**3.92**	**0.15**	**1.54**	**0.80**	**1.86**	**0.11**	**0.10**	**3.42**

HYDRO-ANDRADITE HADR-3	***SiO_2***	***TiO_2***	***Al_2O_3***	***Cr_2O_3***	***$(FeO)_T$***	***MnO***	***MgO***	***NiO***	***CaO***	***Na_2O***	***K_2O***	***TOTAL***
Mean	*35.47*	*1.02*	*3.16*	*0.34*	*18.75*	*0.33*	*3.92*	*0.48*	*29.83*	*0.19*	*0.11*	*93.61*
Standard deviation (1 σ)	*1.69*	*0.72*	*1.35*	*0.14*	*4.25*	*0.13*	*1.92*	*0.95*	*2.62*	*0.14*	*0.09*	*4.66*

Supplementary Table 13. Microprobe analyses (%Wt) and average compositions measured on two type 3-hydroandradites (Hadr-3) in the Casale serpentinites. The analyses reported here were retrieved from one map where two Hadr-3 grains were present. No chemical zonation was observed and thus there is no core/rim distinction for the reported analyses. $(FeO)_T$ corresponds to total ferric and ferrous iron. Similarly to Hadr-2, Cr_2O_3 content is low (~0.3%Wt).

Late serpentine vein

	SiO_2	TiO_2	Al_2O_3	Cr_2O_3	$(FeO)_T$	MnO	MgO	NiO	CaO	Na_2O	K_2O	TOTAL
Late serpentine vein Point 1	42.27	0.00	2.68	0.02	5.30	0.02	35.39	0.21	0.23	0.06	0.00	86.22
Late serpentine vein Point 2	42.57	0.05	3.27	0.01	5.97	0.00	33.73	0.01	0.28	0.04	0.02	85.98
Late serpentine vein Point 3	43.08	0.01	2.87	0.04	4.65	0.00	32.44	0.32	0.31	0.06	0.00	83.93

LATE SERPENTINE	SiO_2	TiO_2	Al_2O_3	Cr_2O_3	$(FeO)_T$	MnO	MgO	NiO	CaO	Na_2O	K_2O	TOTAL
Mean	42.64	0.02	2.94	0.02	5.31	0.01	33.85	0.18	0.27	0.05	0.01	85.30
Standard deviation (1 σ)	0.41	0.03	0.30	0.01	0.66	0.01	1.48	0.16	0.04	0.01	0.01	1.26

Supplementary Table 14. Microprobe analyses (%Wt) and average compositions measured on serpentine from late veins to which the Hadr-3 are associated in the Casale serpentinites. Analyses were performed in punctual mode. $(FeO)_T$ corresponds to total ferric and ferrous iron.

Band number	Wavenumber (cm^{-1})	Tentative vibration assignments
1	993	*Hydro-andradite*
2	-	*Cr^{3+} luminescence*
3	1322	*CH_2 wagging*
4	1342	*CH deformation*
5	1370	*CH_3 in aliphatic compounds (CH_3 symmetric deformation)*
6	1450	*CH_2 and CH_3 in aliphatic chains (bending, scissoring, antisymmetric stretching)*
7	1532	*Aliphatic COOH*
8	1555	*COO^- antisymmetric stretching*
9	1002	*C-C aromatic or phenyl*
10	1400-1420	*COO^- symmetric stretching*
11	1580	*C=C aromatic*
12	1605-1610	*C-C aromatic ring stretching*

Supplementary Table 15: Band positions and vibration assignments for the Raman spectra displayed in ***Fig. 5****. Assignment based on refs (1-8) and Rruff database[9].*

Assemblage	Type of analysis	# Acquisition	Position of CH_2-asymmetric stretching (cm^{-1})	Intensity of CH_2-asymmetric stretching (I_{CH2}, a.u.)	Position of CH_3-asymmetric stretching (cm^{-1})	Intensity of CH_3-asymmetric stretching (I_{CH3}, a.u.)	$R_{CH2/CH3} = I_{CH2}/I_{CH3}$
CCM-HemSap	Ponctual	1	2932.53	33.47	2961.21	29.01	1.15
		2	2927.81	37.96	2957.76	38.38	0.99
		3	2933.14	37.27	2958.99	30.30	1.23
		4	2927.03	61.75	2960.18	52.30	1.18
		5	2929.40	33.95	2960.64	26.50	1.28
CCM-HemSap	Mapping	1	2930.08	19.74	2960.27	16.41	1.20
		2	2928.20	7.66	2961.11	6.77	1.13
		3	2930.60	76.85	2960.90	38.75	1.98
		4	2930.70	52.34	2960.39	23.64	2.21
		5	2930.59	37.41	2959.82	17.57	2.13
		6	2930.41	86.38	2959.66	55.24	1.56
		7	2930.47	72.05	2959.03	43.21	1.67
		8	2930.51	87.13	2959.14	56.03	1.56
		9	2930.19	36.72	2959.82	21.54	1.70
		10	2930.66	62.55	2959.75	29.02	2.16
		11	2930.68	116.01	2959.39	54.53	2.13
		12	2930.47	44.16	2960.31	29.37	1.50
		13	2930.33	51.66	2960.46	33.08	1.56
		14	2930.62	75.60	2959.56	37.52	2.01
		15	2930.55	51.39	2959.74	22.76	2.26
		16	2930.37	60.56	2960.56	21.16	2.86
		17	2928.70	46.92	2959.74	37.36	1.26
		18	2930.42	106.60	2958.20	83.19	1.28
		19	2929.95	67.37	2959.73	34.90	1.93
		20	2929.72	118.90	2959.82	85.30	1.39
		21	2930.25	116.51	2957.50	85.66	1.36
		22	2928.66	14.40	2960.82	9.65	1.49
		23	2928.76	41.05	2961.27	32.17	1.28
		24	2929.80	51.22	2960.98	52.62	0.97
		25	2929.95	104.96	2956.74	107.04	0.98
		26	2930.27	68.74	2960.04	65.72	1.05
		27	2929.22	57.78	2959.64	50.38	1.15
		28	2930.76	51.23	2958.46	48.34	1.06
		29	2929.82	35.90	2959.99	27.62	1.30
		30	2928.89	13.46	2961.20	10.86	1.24
		31	2928.59	49.27	2960.37	41.23	1.19
		32	2929.10	38.07	2960.98	35.77	1.06
		33	2930.10	36.41	2960.16	34.64	1.05
		34	2928.94	20.37	2961.90	17.74	1.15
		35	2927.43	15.28	2961.70	12.91	1.18
					Mean		1.47
					Standard deviation (1 σ)		0.45

Supplementary Table 16. $R_{CH2/CH3}$ ratios and intensities for CH_2– and CH_3–asymmetric stretching vibration bands for the CCM-HemSap assemblages (see main text for acronym definition). FTIR spectra were acquired in punctual and mapping modes.

Supplementary Figures:

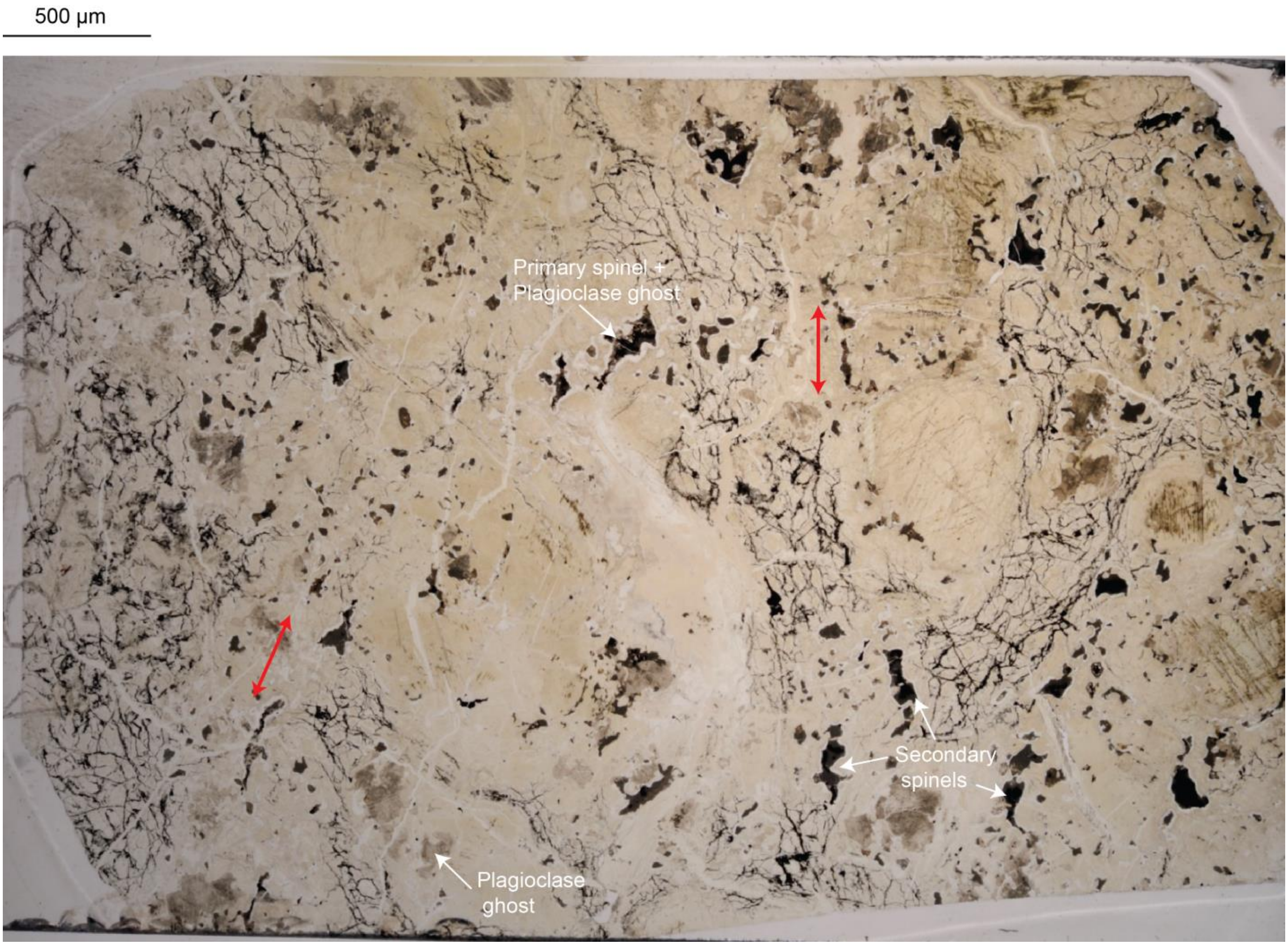

Supplementary Figure 1. Full optical view in transmitted plane polarized light of the studied thin section. The fully serpentinized peridotite of Casale displays few relics of the primary high-temperature paragenesis. The isomorphic mineral substitution allows the identification of the primary assemblage composed of olivine + orthopyroxene + spinel, corresponding to a spinel-field equilibrated harzburgite. No relics of olivine or pyroxene can be found and only few primary spinel cores can still be observed. Melt circulation through the peridotite is highlighted by interstitial secondary spinels and plagioclase ghosts, all aligned along the same direction (indicated by double red arrows), as well as rims of plagioclase ghost around the primary spinels altered in ferritchromite.

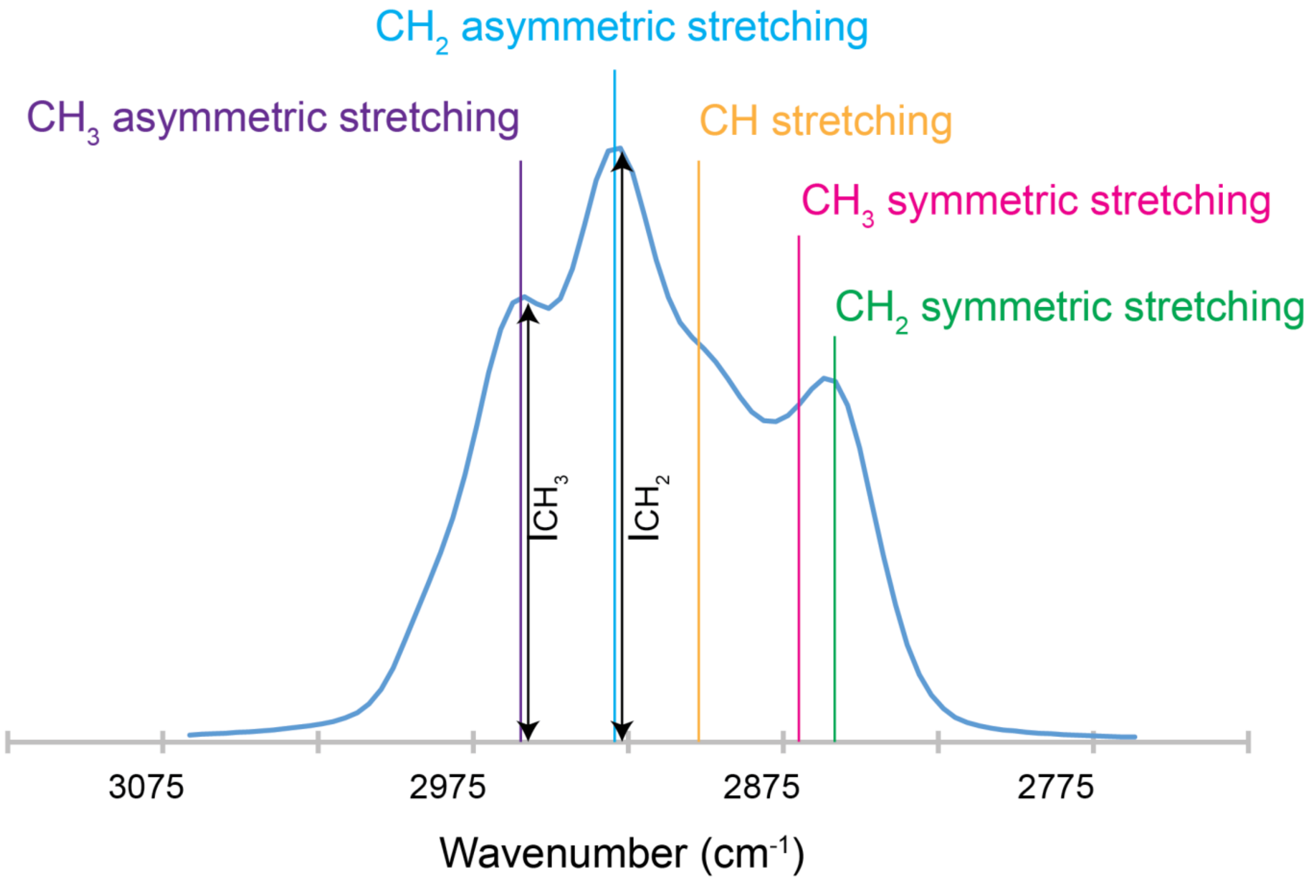

Supplementary Figure 2. FTIR spectrum in the region 3075-2775 cm^{-1} of a CCM-HemSap assemblage (see main text for acronym definition). It shows the presence of an aliphatic component in the associated condensed carbonaceous matter. The position of the different absorption bands constituting the FTIR spectra as well as the intensities (I_{CH2} and I_{CH3}) considered for the $R_{CH2/CH3}$ calculation[10] are reported in ***Supplementary Table 15****.*

Supplementary References:

1. Gaft, M., Reisfeld, R. & Panczer, G. *Modern Luminescence Spectroscopy of Minerals and Materials Ch4. (Springer,* New York, N.Y.*, 2015).*

2. Marshall, C. P., Carter, E. A., Leuko, S. & Javaux, E. J. Vibrational spectroscopy of extant and fossil microbes: Relevance for the astrobiological exploration of Mars. *Vib. Spectrosc.* **41**, 182–189 (2006).

3. Maquelin, K. et al. Identification of medically relevant microorganisms by vibrational spectroscopy. *J. Microbiol. Methods* **51**, 255–271 (2002).

4.. Lambert, J. B., Shurvell, H. F., Lightner, D. A. & Cooks, R. G. Introduction to Organic Spectroscopy. (Macmillan Publishing Company, New York, 1987).

5. Movasaghi, Z., Rehman, S. & Rehman, I. U. Fourier transform infrared (FTIR) spectroscopy of biological tissues. *Appl. Spectrosc. Rev.* **43**, 134–179 (2008).

6. Ivleva, N. P., Wagner, M., Horn, H., Niessner, R. & Haisch, C. Towards a non-destructive chemical characterization of biofilm matrix by Raman microscopy*. Anal. Bioanal. Chem.* **393**, 197–206 (2009).

7. Wagner, M., Ivleva, N. P., Haisch, C., Niessner, R. & Horn, H. Combined use of confocal laser scanning microscopy (CLSM) and Raman microscopy (RM): Investigations on EPS - Matrix. *Water Res.* **43**, 63–76 (2009).

8. Angelena, J. P., Akilan, M., Raja, K., Linet, J. M. & Das, S. J. Growth and characterization of glycinium oxalate crystals in ethanol and methanol. *Optik* **126**, 3912–3916 (2015).

9. Rruff project. Database of Raman spectroscopy, X-ray diffraction and chemistry of minerals. Available at: http://rruff.info/ (2018)

10. Lin, R. & Ritz, G. P. Studying individual macerals using I.R. microspectrometry, and implications on oil versus gas/condensate proneness and 'low-rank' generation. *Org. Geochem.* **20**, 695–706 (1993).
